# Inherited multicentric osteolysis: case report of three siblings treated with bisphosphonate

**DOI:** 10.1186/1546-0096-8-12

**Published:** 2010-04-17

**Authors:** Senq-J Lee, Colin Whitewood, Kevin J Murray

**Affiliations:** 1 Department of Rheumatology, Princess Margaret Hospital, Perth, Australia; 2 Orthopaedic Surgical Department, Princess Margaret Hospital, Perth, Australia; 3 University of Western Australia, Crawley, Australia

## Abstract

Inherited Multicentric Osteolysis (IMO) is an uncommon familial condition of idiopathic pathophysiology causing bone osteolysis and dysplasia. These patients present with common rheumatologic complaints of pain, dysfunction and disability, and are often initially misdiagnosed as a chronic rheumatic disease of childhood such as juvenile idiopathic arthritis. We report a case of three siblings diagnosed with IMO. Diagnosis was made during childhood, with each sibling having different manifestations and course of disease. One had a previous history of bilateral hip dysplasia. Two had osteolysis of the foot, distal tibia and femur (lower limb bones), whilst one had osteolysis of the rib and unusual clavicular fractures. Unusually, all siblings appear to experience decreased pain sensation compared to norms. All siblings were treated with bisphosphonates and experienced a rapid improvement in pain symptoms, decreased analgesic requirements. Two had bone mineral density testing performed and both had increases post-bisphosphonate. In all three, there was subjective evidence of stabilisation of bone disease. Testing for matrix metalloproteinase-2 (MMP2) gene was negative.

## Introduction

Inherited multicentric osteolysis (IMO) is a rare familial skeletal condition characterised by osteolysis leading to bone dysplasia. Patients subsequently experience pain, dysfunction and disability. First described in 1838 by Jackson [1], IMO has been infrequently reported in international literature with synonymous names such as 'vanishing bone' syndrome, inherited osteolysis/arthritis syndromes, multicentric carpal-tarsal osteolysis, nodulosis arthropathy and osteolysis (NAO) syndrome, and Torg-Winchester syndrome [1-3]. The heterogeneity of independent case reports of syndromes sharing similar manifestations and radiologic findings of osteolysis led to grouping by the International Skeletal Dysplasia Society into an "osteolysis" group (Table 1) [4].

**Table 1:** New Nomenclature from Nosology and Classification of Genetic Skeletal Disorders (2006 Revision) Osteolysis group - adapted from International Skeletal Dysplasia Society with permission [4].

Name of disorder	Inheritance	MIM	Locus	Gene	Protein	MIM	Notes
Familial expansile osteolysis	AD	174810	18q22.1	TNFRSF11A	RANK	603499	
Infantile systemic hyalinosis	AR	236490	4q21	CMG2	Capillary morphogenesis gene 2	608041	Incl. Juvenile hyaline fibromatosis (JHF, 228600) and Puretic syndrome
Mandibuloacral dysplasia type A	AR	248370	1q21.2	LMNA	Lamin A/C	150330	
Progeria, Hutchinson-Gilford type	AD	176670	1q21.2	LMNA	Lamin A/C	150330	
Mandibuloacral dysplasia type B	AR	608612	1p34	ZMPSTE24	Zinc metalloproteinase	606480	
Torg-Winchester syndrome	AR	259600 277950	16q13	MMP2	Matrix metalloproteinase 2	120360	Incl. Nodulosis-Arthropathy-Osteolysis syndrome (MIM 605156)
Hadju-Cheney syndrome	AD	102500					
Multicentric carpal-tarsal osteolysis with and without nephropathy	AD	166300					

The disorders grouped in this fashion still had some distinct differences in clinical features, associations, and anatomic locations. Similarities exist between these disorders in that they were familial, and that the primary pathophysiology was of abnormal osteolytic bone causing pain, dysfunction, and disability. In some cases, IMO has other additional clinical manifestations including facial dysmorphism, nephropathy, and neurologic dysfunction. None have been associated with bilateral hip dysplasia.

IMO is often initially misdiagnosed as it mimics other chronic rheumatic disorders such as juvenile idiopathic arthritis [5]. A genetic aetiology is likely, however with the precise genetic mechanisms not yet understood. Studies have shown links of some form of IMO with mutation in gene 16q12-21 which encodes matrix metalloproteinase-2 gene (MMP2). MMP2 deficiency or lack of activity is hypothesised to cause extracellular matrix breakdown, leading to disturbance in bony remodelling favouring osteolysis [1].

Bisphosphonate therapy was commenced in all three children. Bisphosphonates are agents which decrease osteoclastic activity, hence favouring bone formation [6]. Extensive literature supports bisphosphonate use in adults [7,8]. Bisphosphonates are now used in the paediatric population to successfully treat a variety of conditions including osteoporosis and osteogenesis imperfecta. Paediatric case reports suggest that bisphosphonates may have analgesic effects, improve range of movement and function, improve BMD, and potentially improve the natural history of osteonecrosis [9-12]. Some of these studies have shown reduced risk of fractures, but overall data to date is inconclusive. Similarly, data on risk of further surgical intervention is inconclusive.

We present a report of three siblings of a non-consanguineous Caucasian family diagnosed with a possibly unique form of IMO treated with bisphosphonates, seen in our Rheumatology Department of a tertiary paediatric hospital. Consent and permission to publish relevant information, clinical photos, and radiologic images were obtained from the parents of the three siblings.

## Clinical reports

### Case 1

Sibling A is the eldest female sibling of a family who migrated from New Zealand to Australia. Currently 12 years old, she was first seen by our Rheumatology team at age 9 when referral was made from Orthopaedics for management of worsening lower leg disease.

Sibling A was initially seen by an Orthopaedic surgeon during infancy and diagnosed with bilateral developmental dysplasia of the hips, surgically repaired at 17 months of age. At 2 years of age, she presented with a painless deformity of her left ankle. At this stage she was still physically active, participating in ballet. Imaging (X-ray and MRI) revealed osteonecrosis of the left talar body, os calcis, and cuboid. Initial management was with analgesia, rest, casting and splinting with an ankle foot orthosis. Her condition gradually deteriorated over time. At age 4 she developed idiopathic osteonecrosis of her left third metatarsal. Biopsies of the osteonecrotic sites confirmed osteonecrosis with negative bacterial cultures. At age 6 she suffered an atraumatic painless fracture of her right first metacarpal.

**Figure 1:**
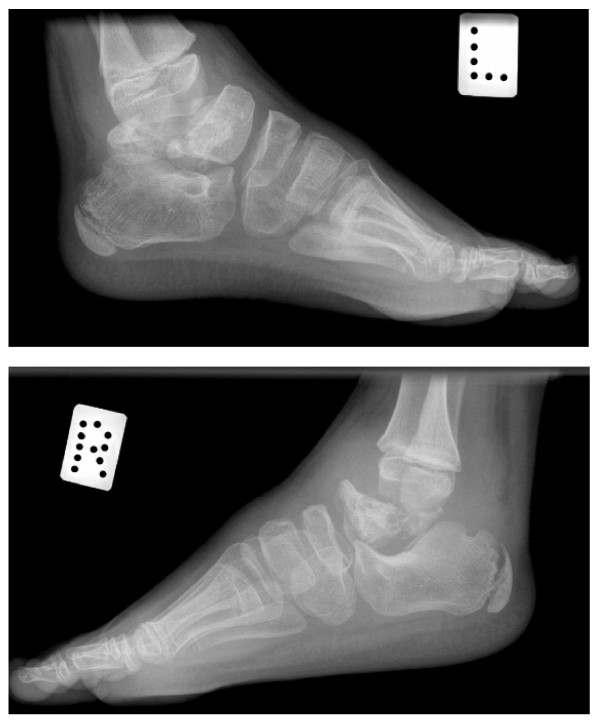
**Sibling A - Fragmentation and destruction of posterior-mid aspect of left talus and right talus, aged 8**.

**Figure 2:**
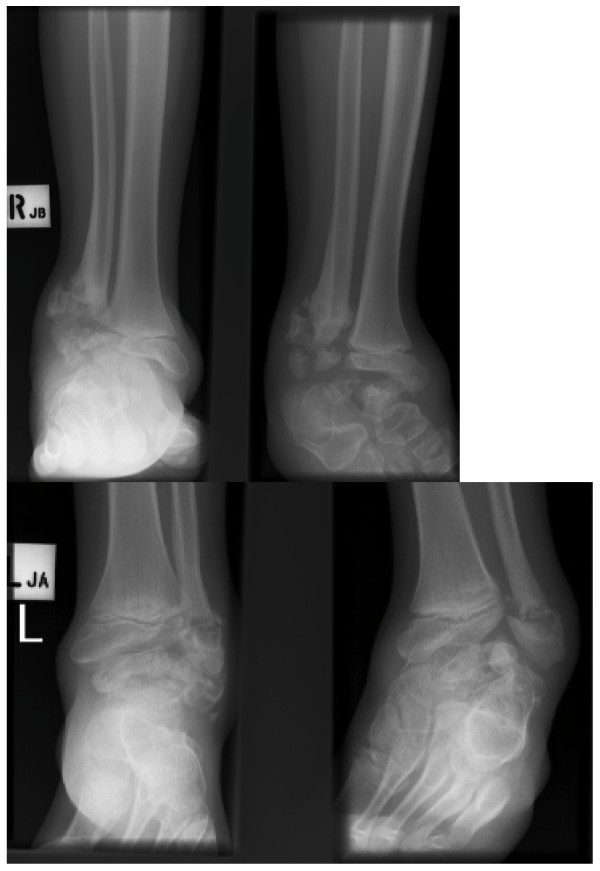
**Sibling A - Bilateral fragmentation and progressive bony destruction of the distal fibula, with tibial sparing**. Disintegration of both tali. Aged 8 1/2 years.

Her younger sister started developing similar problems at this stage and both were referred to a Geneticist and Neurologist for assessment and diagnostic assistance. Differential diagnoses included a familial osteolytic disorder, or possible congenital insensitivity to pain syndrome. Sibling A's disease progressed, involving her right talus at age 7. At this stage she had bilateral lower limb disease [Figure 1, 2].

**Figure 3:**
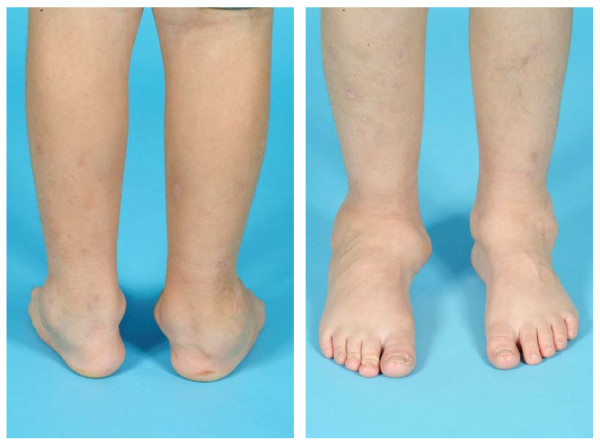
**Sibling A - Clinical photos (posterior and anterior aspects) showing bilateral deformities in both feet and ankles, aged 9**.

Clinical examination on first review by a rheumatologist at age 9 revealed a well child with bilateral gross deformities of her ankles and feet [Figure 3]. There was no dysmorphic features. She had scars on her hips from previous surgical repair of bilateral hip dysplasia. Height and weight were progressing normally for age during early childhood. Developmental examination was normal for age. Neurological examination showed a mild subjective decrease in pain sensation generally, but was otherwise normal. Serologic testing revealed a mild elevation of ESR (20 mm/hr) but FBP U&E ANA CRP were normal. At this stage no biopsies or cultures were performed. Metabolic studies were normal, and nerve conduction velocities revealed intact motor function but borderline delayed sensory function. Testing for mutation of MMP2 gene was negative.

Initial discussions led to a favoured diagnosis of hereditary sensory and autonomic neuropathy (HSAN), however we felt that a more accurate diagnosis was IMO given her disease presentation and clinical progression, later assisted by investigation findings of multiple aseptic osteolytic lesions. The mode of inheritance was thought to be autosomal recessive as generations of the family were not affected. Treatment was initiated with regular non-steroidal anti-inflammatory medication (NSAID) with good analgesic effect.

**Figure 4:**
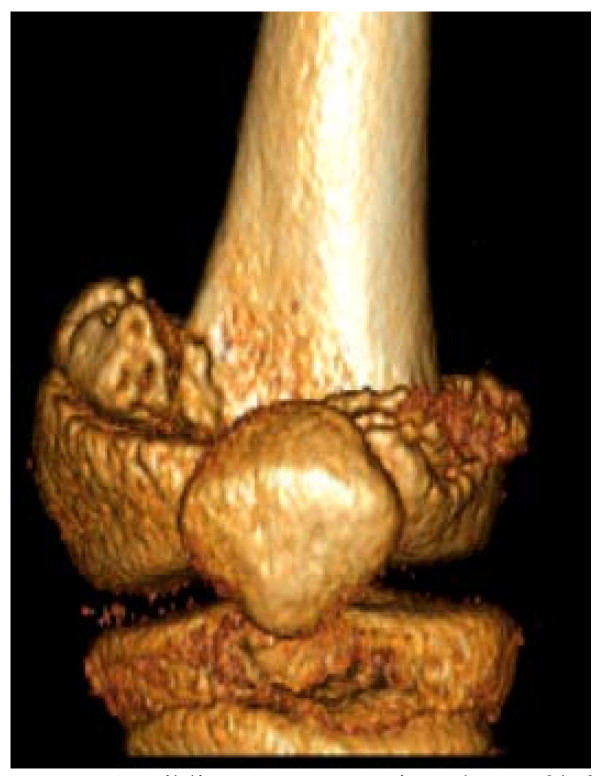
**Sibling A - Reconstituted CT of left distal femur collapse/impaction, aged 10**.

Six months later she developed a Staphylococcus Aureus septic arthritis of her right ankle, and was treated with IV Benzylpenicillin and oral Rifampicin. At age 10 she was diagnosed with an atraumatic impaction or collapse of her distal left femur, with multiple areas of osteolysis surrounding the fracture [Figure 4].

Complications followed - firstly non-union of her fracture, treated conservatively with immobilisation and non weight bearing. She then developed S. Aureus osteomyelitis of her left knee lesion, again treated successfully with a course of IV Flucloxacillin and oral Rifampicin. ESR and CRP were significantly elevated during periods of infection. ESR remained persistently mildly elevated (20 to 30 mm/hr) during the course of disease.

Bisphosphonate therapy for IMO was commenced as there were concerns about the non-union of her left distal femoral fracture, gradually worsening osteolysis, and potential complete fragmentation of the distal femoral condyles. She was treated at age 10 1/2 years old with intravenous Pamidronate at an initial dose of 0.5 mg/kg, increasing to 1 mg/kg for following doses. Treatment was performed monthly for six months. Oral calcium and Vitamin D supplements were also commenced. Bisphosphonates provided some apparent benefits including an acute improvement in pain, however there was no difference in range of motion and function. Impact on disease progression was difficult to assess. Following bisphosphonate therapy, there was no improvement in existing osteolytic disease, but there was gradual healing and union of her left distal femoral fracture over time. To date she has not had any new fractures, and no new focal areas of osteolysis. Bone mineral density improved, with whole body BMD improving from 0.66 (z score -2.56) to 0.96 (z -0.1) after 12 months. Similarly, L2-L4 lumbar BMD scores improved similarly, from 0.57 (z -0.59) to 0.99 (z +0.6). Left femoral head BMD scores improved from 0.43 (z -3.01) to 0.81 (z -0.7). Only a minor transient self-resolving fever was observed during the infusion. There were no other reported side effects.

**Figure 5:**
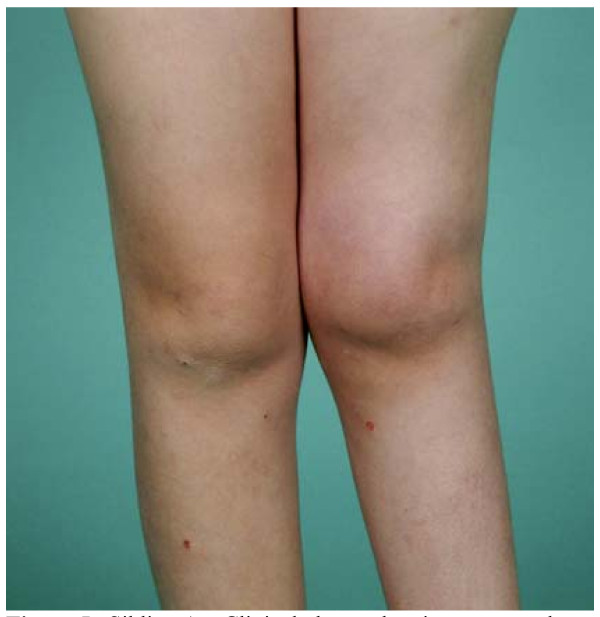
**Sibling A - Clinical photos showing severe valgus of her left knee and shortening of femur, aged 10 1/2**.

**Figure 6:**
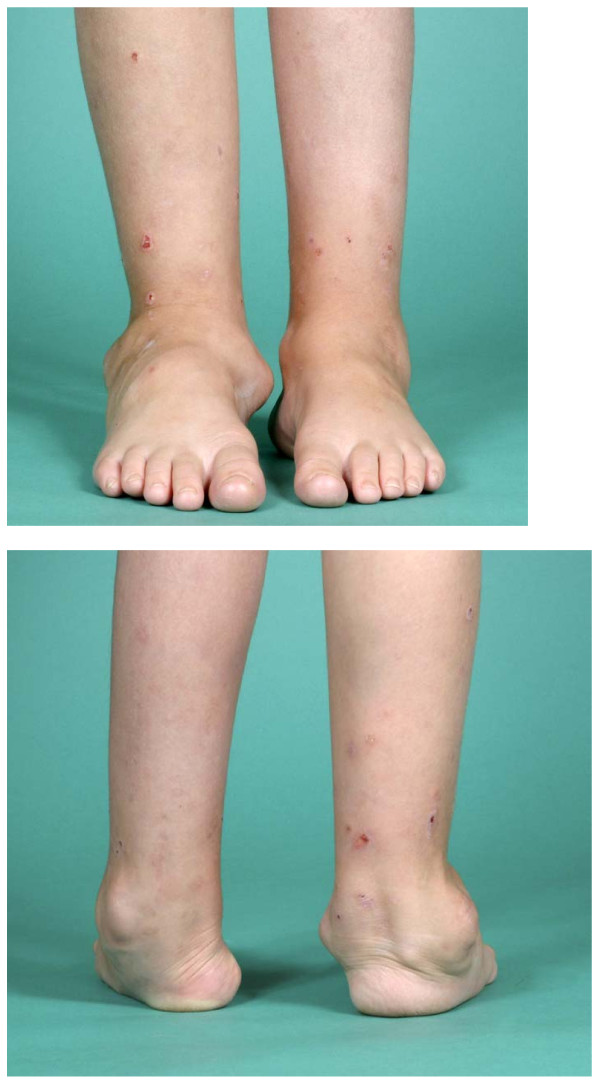
**Sibling A - Clinical photos (anterior and posterior aspects) showing deformities of both ankles and feet, aged 10 1/2**.

Post-bisphosphonate therapy, she had one surgical procedure performed. As a consequence of her left distal femur fracture, she developed significant leg length discrepancy and severe valgus [Figure 5, 6]. Staples were inserted into the medial aspect of her left distal femoral growth plate and bilateral aspects of her right distal femoral growth plate, with the aim of arrest femoral growth and correcting her leg length discrepancy.

### Case 2

Sibling B was the middle male child of the three siblings. He initially presented in clinic only as part of follow-up with the other two siblings. Initial x-ray screening for disease did not find any hip or lower limb bone disease. He suffered his first bilateral clavicular fracture at age 7 after falling whilst playing football. Unusually, he reported feeling some initial pain but continued to play football after sustaining the fractures. These fractures were managed conservatively, and healed slowly with large callous formations at each fracture site. Following this, he had recurrences of clavicular fractures thereafter.

**Figure 7:**
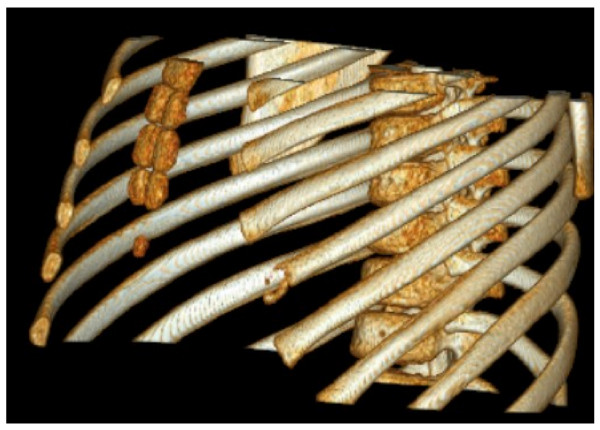
**Sibling B - CT Chest showing osteolysis of the left 6^th ^rib, aged 10 1/2**.

At age 10 1/2 he presented to the Emergency Department complaining of left chest pain. History revealed he suffered minor trauma to his chest after falling off a treadmill. Given the family history, a CT chest was performed revealing an osteolytic lesion of the left 6^th ^rib anteriorly [Figure 7].

Serology revealed normal CRP, FBP, Ca, PO4, Vit D and ALP. ANA and ESR were not performed. Bone densitometry testing via dual-energy x-ray absorptiometry was normal for age. Oral Alendronate 40 mg twice weekly was subsequently commenced, as well as calcium and vitamin D supplements.

Sibling B experienced an improvement in pain scores following oral bisphosphonate therapy. Both clavicular and rib lesions reduced in size clinically. Range of motion and function assessment was not applicable. At the time of writing, duration of treatment with bisphosphonates is 3 months, hence it is too early to fully analyse other outcomes and effects from bisphosphonate therapy. No adverse effects from therapy were reported.

### Case 3

Sibling C was the youngest female of the three siblings. She had been concurrently reviewed at the same times as both elder siblings. Onset of clinical findings suggestive of IMO occurred at age 3 when she developed swelling of her right foot. X-rays of her right foot revealed an irregular broad distal tibial epiphysis and metaphysis, bony sclerosis in the tarsus, and avascular changes of her cuboid. She remained largely asymptomatic at this stage with little progression.

**Figure 8:**
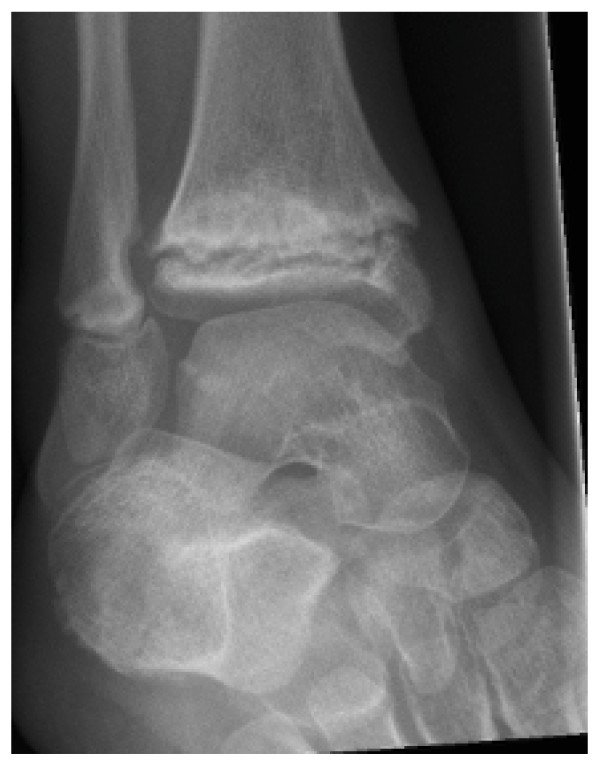
**Sibling C - X-ray of right ankle showing abnormal osteolytic process of epiphyses of distal tibia and fibula, aged 8 yo**.

At age 8, clinical examination and investigations showed that her right ankle osteolysis was slowly progressing [Figure 8]. A six-month course of IV Pamidronate 1 mg/kg monthly was commenced at this stage as a prophylactic measure aiming to impede further disease progression. Oral calcium and vitamin D supplements were also commenced. Bisphosphonate therapy provided some analgesic benefit to her right foot, but no difference was found in range of motion of her right ankle. Whole body BMD improved from 0.81 (z-score -0.1) to 0.92 (z +1.1) after 12 months. L2 to L4 lumbar BMD improved from 0.72 (z +0.2) to 0.86 (z +1.4). Femoral head BMD increased from 0.71 to 0.89 (no z scores reported). During bisphosphonate therapy, she developed swelling of her left 5^th ^toe, and pain in her left ankle. X-rays revealed a pathologic fracture and lucency of the left 5^th ^toe. X-rays of her right ankle did not reveal any change of her abnormal epiphyseal osteolytic process of tibial and distal femoral epiphyses. Treatment consisted of non weight-bearing, followed by partial weight bearing with a walker for ambulatory assistance. Serology including FBP, ESR and CRP were normal.

Post bisphosphonate therapy, she had no further radiologic or clinical deterioration, with stabilisation of existing osteolysis on radiologic imaging.

**Figure 9:**
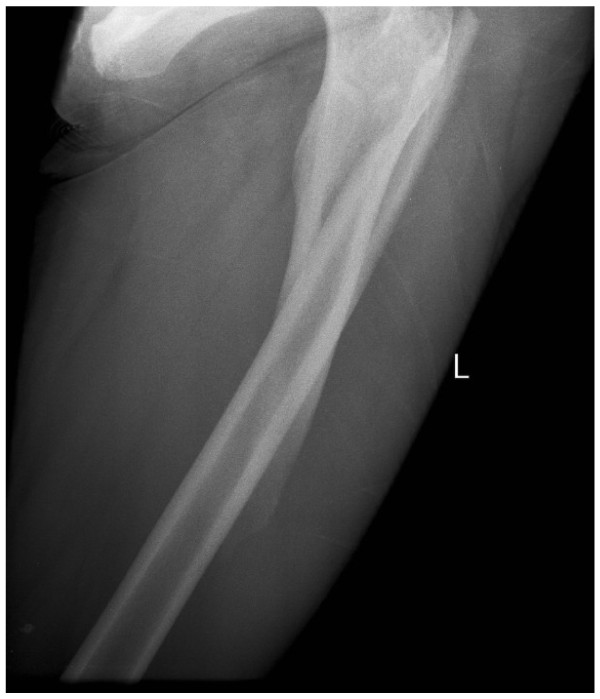
**Sibling C - recent spiral fracture of her left proximal femoral shaft, with no history of significant trauma**. Note significant displacement and shortening.

At age 9, six months after cessation of Pamidronate therapy, she suffered an atraumatic spiral fracture of her left proximal femoral shaft [Figure 9]. The fracture was treated with traction. We continue to follow her progress.

Sibling C experienced mild abdominal pain as an adverse effect of bisphosphonate therapy, however this was transient and did not require treatment. There were no other adverse effects reported. We have successfully applied for extension of bisphosphonate therapy with IV Zoledronic acid for both sibling A and sibling C and await clinical outcomes.

## Discussion

IMO is an interesting yet rare familial osteolytic condition of largely unknown aetiology. The International Skeletal Dysplasia Society recently revised their nosology and classification guidelines in which IMO encompasses many different osteolytic syndromes. These syndromes have some overlap of clinical features but commonly share the same pathogenesis of osteolysis, with a pattern of familial inheritance. Clinical symptoms or signs usually occur early in childhood (usually 2^nd ^or 3^rd ^year of life). Findings include multiple bone and joint pain and bony "dysplasia" with associated decreased range of movement and function. Diagnosis is usually made with characteristic osteolytic changes on radiological investigations. Serologic and histologic investigations are always negative for classical inflammatory or autoimmune causes. Further investigations must always be initially performed to distinguishing IMO from differential diagnoses, including inflammatory, autoimmune, endocrine and metabolic causes. As the pathophysiology is unknown, differential diagnoses of IMO must include conditions which cause avascular necrosis. Treatment of IMO with traditional anti-inflammatory or anti-rheumatic medications appears to have little effect on pain or disease progression. Genetic testing have revealed possible associations with specific genes such as MMP2, however sibling A was tested and found not to have a mutation in MMP2. Our IMO patients may be a unique form genetically, as it is unusual that these siblings experienced decreased sensation to pain. One sibling had hip dysplasia, which is not a characteristic of the other osteolytic syndromes. In this family, mode of inheritance was thought to be autosomal recessive as no other generations were affected.

Bisphosphonates are analogues of inorganic pyrophosphate with selectivity to bone rather than other tissue. Their primary mechanism of action is to either inhibit osteoclastic activity, reduce osteoclastic recruitment, differentiation and resorptive activity, or induce apoptosis [6], thereby favouring bone formation. Evidence in paediatric use suggests potential benefits, which may include reduction in pain, increased function and range of motion, improved BMD, reduced risk of fractures, and retardation of the natural disease history.

In this case report, we have found that bisphosphonates in these children with IMO have been beneficial in improving acute pain and BMD. There was no effect on range of motion. In two siblings, there was radiologic evidence suggesting that bisphosphonates may retard the natural history or progression of the disease. We could not comment on the effects on fracture risk as one sibling developed a significant fracture post-bisphosphonate, even following an improvement in BMD. Similarly, we could not comment on whether bisphosphonates reduce the risk for further surgical intervention as one sibling required stapling to correct leg length discrepancy. Bisphosphonate therapy in these three siblings seemed safe, as reported side effects were mild and clinically insignificant. Given that the treatment options for conditions such as IMO are limited, we conclude that bisphosphonate therapy could be considered for treatment of IMO as it appears safe and has potential benefits as described above.

## Conclusion

IMO is a rare familial condition which must be considered in children and adolescents with idiopathic osteolysis of bones affecting other members of the family. The pathophysiology is thought to be of genetic aetiology but not well understood. Reported genetic links include mutations of the MMP2 gene. IMO often mimics the symptoms of other chronic rheumatologic disorder, and is often initially misdiagnosed. It is a diagnosis of exclusion, hence investigations must be performed to rule out other diagnoses. Patients with IMO may benefit from bisphosphonate therapy. These benefits may include pain relief, decreased analgesic requirements, improved BMD, and potentially reducing new fracture or osteolytic collapse risk. Given small numbers seen in any single tertiary centre, further multi-centre studies into safety and efficacy of bisphosphonate therapy in conditions such as IMO are warranted.

## Abbreviations

MRI: magnetic resonance imaging; ESR: erythrocyte sedimentation rate; FBP: full blood picture; U&E: urea, electrolytes and creatinine; CRP: C-reactive protein; ANA: anti-nuclear antibody testing; IV: intravenous; CT: computed tomography imaging; Ca: calcium; PO4: phosphate; Vit D: vitamin D; ALP: alkaline phosphatase.

## Consent

Written informed consent was obtained from the patient for publication of this case report and any accompanying images. A copy of the written consent is available for review by the Editor-in-Chief of this journal.

## Competing interests

The authors declare that they have no competing interests.

## Authors' contributions

SJL and KJM both participated in conceiving and designing the case report. SJL acquired all relevant clinical information, photos and images. SJL drafted the manuscript. CW and KJM assisted in critical revision of the report. KJM assisted SJL in final revision to be published. All authors read and approved the final manuscript.

## Authors' informations

SJL is an advanced trainee in Paediatric Rheumatology, governed under the Royal Australasian College of Physicians. CW is a consultant Orthopaedic Surgeon and a member of the Royal Australasian College of Surgery (Orthopaedics). KJM is a consultant Paediatric Rheumatologist and a member of the Royal Australasian College of Physicians.
